# Understanding the Characteristics of At-Risk Youths in Guatemala: Evidence from a Sports for Human Development Program

**DOI:** 10.3390/children10010134

**Published:** 2023-01-10

**Authors:** Pedro Danilo Ponciano Nuñez, Iago Portela-Pino, María José Martínez-Patiño

**Affiliations:** 1 Research Group on Education, Physical Activity and Health (GIES 10) Galicia Sur Research Institute (ISS Galicia Sur), SERGAS-UVIGO, 36312 Vigo, Spain; 2 Department of Physical Education, Sport and Leisure, Universidad del Valle de Guatemala, Guatemala City 01015, Guatemala; 3 Department of Health Sciences, Faculty of Health Sciences, Isabel I University, 09003 Burgos, Spain; 4 Olympic Studies Centre, Faculty of Science, Education and Sport, University of Vigo, 36005 Pontevedra, Spain

**Keywords:** sports, health literacy, physical literacy, social–emotional skills, life skills

## Abstract

Guatemala is a multiethnic and multicultural country that has suffered from poverty and violence. Sports can serve as tool to foster development across the country; however, there is limited research on the use of sports as a tool for promoting broader social benefits in Guatemala. The purpose of this study was to compare sports and the health and physiological characteristics of at-risk youths in Guatemala. The research objectives were achieved through a quantitative approach and the participation of 90 youths involved in an educational organization through sports and 91 youths who have not been influenced by any organization. The results showed that urban at-risk youths involved in a sports for education organization develop more self-esteem; they have higher levels of physical activity than their peers who are not involved in an educational organization; the socioemotional competencies of self-regulation and motivation are higher in urban areas; empathy is higher in men than in women; the level of the self-perception of health is lower and health literacy higher. However, the at-risk youths who are not involved in an educational organization showed that their self-regulation was higher, and the level of health literacy was higher for all factors. This was through a set of attitudes and skills as a result of their historical development and sociocultural strategies transmitted from generation to generation to foster health and physical activity.

## 1. Introduction

Adolescence is considered a transition period of physical and psychological changes [1]. This transition period should occur with a good balance of body and mind development [2]. During this transition period, youths experience a variety of insecurities and choices [3]. Adequate development of the social competences that allow them to make the best decisions according to the needs of their daily lives is fundamental [4,5,6]. Sports can become a platform to foster health [7,8] and enable broader positive social outcomes (e.g., inclusion, life skills) in communities [9,10,11,12]. For example, the participation in sports-based programming of at-risk youths has demonstrated a greater impact on their lives than music or art programs [13]. This has led to positive psychosocial and peacebuilding outcomes in countries with a long history of conflict [14,15,16]. Youths are considered “at-risk” when it is difficult for them to participate in their social and educational settings [17].

Sports-based intervention programs appear to improve intellectual development and civic values [18]. Youths who participate in physical activity have improved mental health [19] and experience an increase in their self-esteem [20]. Social backgrounds are affected by crime and violence [21], which result in hooliganism and corruption [16,22,23], due to the need for acceptance by peers [24]. The establishment of sports-based programming can result in beneficial peer interactions and have a positive influence on self-esteem [10,25]. The improvement of self-esteem decreases the risk of acquiring depression by solving problematic situations in the social environment [26]. This leads to the need for long-term programs based on evidence [10,16,27] for the improvement of the psychological and physical characteristics [28] of youths, who then become community role models [29,30,31,32].

Systematic programs can contribute to eradicating school truancy, which results in low educational achievement, family disintegration, and less sports participation [33]. School departees suffer from deficient health, causing them to regularly go to the doctor and not participating or being able to participate in heavy physical activity [34]. Youths who participate in physical activity programming drop out of school less often, and their self-esteem levels continually increase [10,35]. Sports programming can serve as space for the detection of sexually transmitted diseases (STDs), as an organization is required to have access to participants’ previous health screening before joining [36]. However, the power of sports (including observable impacts) has been challenged by the scholarly community [37], partly due to the limited evidence of the types of outcomes of these interventions [38,39]. Critical thinking is necessary to question what conditions are required and to explain how change happens using sports [38,40]. The positive impact depends on various factors (e.g., context, type of sports, role models) [41,42] because sports does not promote positive outcomes by itself [15,16,22,23].

Guatemala is a country with social inequality (e.g., education, violence, and delinquency) [43], high emigration rates [44], a high number of homicides [21], and conflict between local ethnic groups [45], and due to this, describing the characteristics of local youths becomes relevant [43]. The purpose of this study was to compare the sports, health, and physiological characteristics of at-risk youths from urban and rural settings in Guatemala. This study establishes a criterion for the current development of these characteristics in the participants. The participants from the urban region are exposed to the implementation of a values education curriculum through sports and belong to a youth foundation [43]. The Guatemalan Olympic Foundation (FUNOG) pursues the positive development of youths who live in highly dangerous neighborhoods of Guatemala City and who have mostly moved from rural to urban areas [21,43,46]. On the other hand, the youths from the rural region do not participate in an organization and are not exposed to a values education curriculum through sports. This study will establish if at-risk youths who are part of an educational program show better physical, educational, and health characteristics compared to their peers who are not part of a program. 

## 2. Materials and Methods

Based on the objective, an exploratory case study with a descriptive characteristic utilizing purposive sampling was chosen to allow the authors to make decisions adjusted to the characteristics of the participants. All the youths participating in the sports-based educational organization were part of the research study. The program analyzed was implemented by FUNOG’s administrative staff and trainers. Participants must live in a neighborhood where the organization operates and join voluntarily with the permission of their parents. The authors intentionally identified another population of at-risk youths with similar characteristics (e.g., age, vulnerable condition) from another region of the country who do not participate in a sports education organization to recover the data and finally make a comparison based on the descriptive analysis of the main variables. The study used scales commonly applied in educational and psychological studies, pointing out the internal consistency of each of the scales. 

### 2.1. Study Context

The participants of the urban region (Guatemala City, Guatemala) are part of the Guatemalan Olympic Foundation (FUNOG), an educational program based on Olympic values that promotes physical activity as a space for social transformation [21,43,46]. Recent studies have identified that the sports used by FUNOG respond to a pedagogical strategy that seeks to establish safe spaces to transform social scenarios outside of traditional sports (e.g., soccer, basketball) that young people are accustomed to practicing, allowing them to have access to other sports where they can change their mindset by experiencing other social environments (e.g., racquet sports, swimming, gymnastics, rugby) [43]. The program involves different stakeholders for its development in the field (e.g., parents, coaches, athletes, and volunteers) and financial sustainability (national and international organizations). The program includes two scholarship initiatives that provide the participants with educational opportunities and a tutoring system to monitor homework completion, favoring the reduction of the school truancy rate of young people who participate in an educational organization [43]. The rural region was represented by youths who are not involved in an educational organization from the Highland community of Momostenango (Totonicapán, Guatemala). These youths were purposely selected based on similar risk characteristics as youths who participate in an educational program and voluntarily participated in the study through the support of a local leader who facilitated contact with at-risk youths inside the community.

### 2.2. Participants

The study consisted of a sample of 181 participants (N = 91 urban; N = 90 rural). The sample of participants were 50.8% (N = 92) male and 49.2% (N = 89) female. The median age was 14.44 (max. = 18; min. = 12).

### 2.3. Questionnaires

The application of the research instruments consisted of two phases. Phase I was a purpose-built survey that included personal and familiar variables for each participant. Phase II was the use of questionnaires to measure the level of physical activity, social–emotional competences, self-esteem, purpose in life, and health literacy. The questionnaires were the following: (i) Assessment Scale of Social-Emotional Competences (ASSEC), (ii) Physical Activity Questionnaire for Adolescents (PAQ-A), (iii) Rosenberg Self-Esteem Scale (RSE) and (iv) Health Literacy Survey Questionnaire (HLS-EU-Q47).

The Assessment Scale of Social-Emotional Competences (ASSEC) was used to measure the level of progress in several social–emotional competences: (i) self-regulation, (ii) teamwork, (iii) self-awareness, (iv) interpersonal regulation, (v) empathy, (vi) motivation, and (vii) basic conflict resolution [47]. The internal consistency in the study was 0.90.

The Physical Activity Questionnaire for Adolescents (PAQ-A) assesses the amount of physical activity of youths of 14 to 20 years of age. The questionnaire is a Likert scale that uses nine items to evaluate the levels of physical activity that the participant has carried out in the last seven days, and its final result is the mean of the points for Items 1–8 [48]. Item 1 refers to any physical activity carried out in the participants free time. It includes 24 sub-items, and 22 are adapted to the activities practiced in Spain [49], while the last two are completed by each participant and can include a maximum of two physical activities not offered by the questionnaire. The points for Item 1 are obtained from the average of all the sub-items. Item 2 measures the amount of physical activity that the participants performed through a physical education class. Item 3 shows the level of physical activity before and after a meal. Item 5 measures how many times the participant was active after school until 6:00 p.m. seven days prior to the use of the questionnaire. Additionally, Item 5 measures how many times the participant was active between 6:00 p.m. and 10:00 p.m. Item 6 evaluates the number of times that the participants were active on the weekend before applying the questionnaire. Item 7 shows the frequency and intensity of the physical activities of the last week carried out by the participants. Item 8 includes seven sub-items for each of the seven days of the week. The participants mark the frequency with which they perform physical activities, and the final score is the total of the mean of the sub-items. Item 9 is used to establish the less-frequent types of physical activity due to a disease; this is not included in the calculation of the final score. The score is established between 1 (low physical activity index) and 5 (high physical activity index). Three ranges are used to show PAQ-A results ≤ 2 as “low activity”, >2 and ≤3 as “moderate activity”, and >3 as “high activity” [50,51]. 

The Rosenberg Self-Esteem Scale (RSE) is used (Spanish adaptation) to measure the level of self-esteem of the participants [52]. The scale includes ten items on a Likert scale: (i) strongly agree, (ii) agree, (iii) disagree, and (iv) strongly disagree. The self-esteem score is categorized as follows: low self-esteem (<25 points), average self-esteem (26–29 points), and high self-esteem (30–40 points). The Purpose In Life test (PIL) is used (Spanish adaptation) for evaluating the purpose in life based on the original test of Crumbaugh and Mabolick, which includes 20 Likert-scale items with answer options from 1 to 7 [53]. Life purpose is an empirical construct supported by multiple studies [53] consisting of four factors: (i) perception of purpose (FPI) including the knowledge and motivations a person perceives in order to live and value his or her own life (Items: 4, 6, 9, 10, 11, 12, 16, 17, and 20); (ii) experience of purpose (FP2) evaluates the participant’s perception of personal existence in relation to good things, such as everyday experiences and events (Items: 1, 2, 5, 9, 17, 19, and 20); (iii) goals and tasks (FP3) evaluates objectives linked to concrete actions in life and the responsibility assumed by setting such objectives (Items: 3, 7, 18, 13, 17, 19, and 20; (iv) fate–freedom dialectics (FP4) considers the dilemma between freedom and fate in an individual´s life and contemplating death as an inevitable end (Items: 14, 15, an 18) [53,54]. The internal consistency in the study was 0.78. 

The Health Literacy Survey Questionnaire (HLS-EU-Q47) measures how participants obtain, understand, value, and apply information to make decisions related to disease prevention and health promotion [55]. The HLS-EU-Q47 includes 47 items in 12 sub-categories, each of them covering three different areas: (i) attention and care, (ii) disease prevention, and (iii) health promotion [56], and it provides the information of four different abilities in health literacy, gaining access, understanding, evaluating, and applying. The instrument is marked on a Likert scale based on four categories: (1 = very difficult, 2 = difficult 3 = easy, 4 = very easy). To identify risk factors of deficient health literacy (HL), the participants’ scores were classified as HL deficient: <25.88 and HL excellent > 41.45 [56]. The internal consistency in the study was 0.94.

### 2.4. Data Analysis

For the descriptive calculations, measures of central tendency such as the mean, standard deviation, and minimum and maximum of all the scale variables were used, as well as asymmetry and kurtosis. The Kolmogorov–Smirnov test (*p* > 0.05) was used to verify the parametric assumption of normality. Three of the samples reported non-parametric distributions: self-esteem (K-S = 0.005), prevention (K-S = 0.021), and conflict resolution (K-S = 0.015). Since the other variables met the conditions of a normal distribution, we proceeded to calculate the parametric statistics for all the hypotheses. To establish the relationship between the quantitative cut-off variables, the Pearson correlation statistic was used in order to establish the difference in the means between the different dependent variables and the categorical polytomous variables (academic qualifications, source of income, religion, residence). The statistical analyses were performed with the SPSS v. 23 statistical program (IBM Corp., 2012). The level of significance for all analyses was *p* < 0.05.

### 2.5. Procedures

The study was regulated based on the Declaration of Helsinki (Hong Kong, September 1989) and the EEC Good Clinical Practice Recommendation (Document 111/3976/88 July 1990). The process strategy was to apply the test to the participants in Guatemala City and Momostenango. The staff (coaches and teachers) were trained by the researchers in the use of the instruments, and their doubts related to the research process were clarified. Staff described to the participants’ parents the conditions, emphasizing that testing would be anonymous and the data of the youths participating would be kept confidential. A consent form was provided and signed by the parents of the participants, and the test was applied by the staff.

## 3. Results

### 3.1. Personal, Familial, and Health Profile of Participants

The participants (N = 181) were categorized in two categories: (i) urban and (ii) rural. In the urban area (participating in the educational organization), 56% were male and 44% were female. In the rural area (not participating in the educational organization), 45.6% were male and 54.4% were female. With respect to family income (Table 1), there were significant differences between the family income of participating and non-participating youths (Chi-squared = 0.0001).

The participating youths having a monthly income of less than EUR 500 made up 61.5% and 36.3% having between EUR 501 and EUR 1000, and 2.2% received more than EUR 1001. The non-participating youths having a monthly income of less than EUR 500 were 6% and 72% having between EUR 501 and EUR 1000, and 12% received more than EUR 1001.

There were 61.9% of participants having completed primary school, 37% secondary school, and 1.1% with no formal education. The employment condition indicated that 86.2% of the participants were not employed, 6.6% worked in the formal economy, and 7.2% worked in the informal economy. There were 96.7% of participants having no vocational training, and 3.4% of the sample had vocational training. Their income showed that 34.3% of the participants received an income through employment and 58% from their families, and 7.7% had no income. The data showed that 70.7% of the participants were Catholic, 16% Christian, 8.3% Evangelical, and 5% non-religious. The mothers of 61.3% of the participants completed secondary school and 23.2 primary school, while 6.1% had employment training, 8.3% technical training, and 1.1% no education. The fathers of 58.6% of the participants completed secondary school, 18.2% received employment training, 12.2% received technical employment training, 10.5% completed primary school, and 0.6% had no education.

### 3.2. Physical Activity, Health, and Psychological Characteristics

The results showed (Table 2) that the average sports, health, and psychological variables of at-risk youths in Guatemala were low. Self-esteem barely exceeded 21 points; the level of physical activity reached an average of 2.87, the three dimensions of health literacy, health care, disease prevention, and health promotion were also low; the mastery of socioemotional competences was medium, except for teamwork, empathy, and self-regulation, which were very low.

Explaining the differences (Table 3) in the sports, health, and psychological variables, all the variables were significant, except for health prevention, self-awareness, conflict resolution, teamwork, empathy, and general socioemotional competence. Self-esteem, physical activity level, interpersonal regulation, and motivation were higher in the urban environment. However, the three dimensions of health literacy were higher in rural areas, as well as self-regulation. With respect to size, the effect was high for almost all variables except for some of the socioemotional competencies.

There were no differences with respect to sex in any of the dependent variables analyzed in either the urban or rural setting, except for empathy in the urban setting ($\bar{x}$♂ = 2.70; $\bar{x}$♀ = 2.12; sig. = 0.037). In the urban setting the level of self-perception of health was negatively related to the dimensions of health literacy (care R = −0.462; prevention r = −0.345, and promotion = −0.433; AS = −0.393).

Among the youths participating in the educational organization in the urban center, 51.6% had been hospitalized once, 36.3% twice, and 12.1% three or more times. Seventy percent had been to the emergency department once, 26.4% twice, and 3.3% three or more times. Among young people living in rural areas who were not involved in the educational organization, no one had ever been hospitalized, and 74.4% had been to the emergency department once and 25.6% twice. No one had ever been hospitalized three or more times. Regarding self-perception of health, among young people participating in the educational organization, no one had a poor or poor perception of their health, 28.6% had a good perception of their health, and 71.4% had a high perception of their health. Among youths who were not involved in the educational organization, 1.1% had a poor self-perception of their health, 4.4 poor, 21.1 good, and 73.3% high. Therefore, there were no differences in self-perception of health (Chi-squared = 0.107). Regarding the physical exercise of the youths who were involved in the educational organization, 5.5% never exercised, 18.7% exercised occasionally, and 75.8% exercised frequently. With respect to the youths who were not involved in the educational organization, 2.2% never performed physical activity, 43.3% occasionally, and 54.4% frequently. Table 4 shows the frequencies and significant Chi-square results that demonstrate the association between the different categorical variables.

In both rural and urban areas (Table 5), the age of the children correlated positively, significantly, but lowly with health care, so it seems that the older the child, the higher the health literacy in this dimension is. Self-esteem correlated with the level of physical activity, but with none of the health variables nor with the socioemotional competencies, neither did the level of physical activity or the dimensions of health literacy or socioemotional competencies. As would be expected, the factors of the same scale correlated highly and positively with each other, except for interpersonal regulation with motivation, self-awareness, and conflict resolution, and the same occurred with motivation, self-awareness, and conflict resolution.

## 4. Discussion

The purpose of this study was to compare the sports, health, and physiological characteristics of at-risk youths from urban and rural settings in Guatemala. The study established a criterion for the current development of these characteristics in the participants. The following categories are presented based on the data analysis.

### 4.1. Participant Landscape

Being a multiethnic (Mayan, Garifuna, Xinca, and Ladino) country, Guatemala is composed of twenty-five cultural groups regarding the historical social development of Guatemalans [57]. The youths participating in this study came from urban and rural areas; however, most of the participants who participate in the educational organization were from rural areas nationwide [43]. This is caused by the displacement to the city because of the high rates of unemployment and limited academic training, as it is more important to work to satisfy their basic needs as result of their precarious living conditions [45]. The income of families in rural areas is much higher than youths living in urban areas because the poorest families that come from the rural areas can be residents of the urban areas of Guatemala City due to the need to improve their living conditions, and by residing in prioritized neighborhoods, they can participate in the organization. The participants reported that their income comes mostly from the employment of their parents, and a few of them had no formal income because of not having sufficient academic training, which can cause them to become involved in criminal activities and other antisocial behaviors to satisfy their needs. The participants reported having completed two levels of education, and only a few of them did not have any formal education. This correlates with the evidence of a previous study that showed that most of the at-risk youths are not employed, while others work in formal or informal economies as a result of their poor academic background [57]. Most of the participants did not have vocational training since the institutions in charge request certain academic requirements, which means that their chances of integrating into formal employment will be difficult in the future by already limiting their access to better life opportunities. The academic background of the participants’ parents suggests that at least they had access to one educational stage or technical training to become part of the formal or informal economy. The participants’ religious beliefs showed that Catholicism was the dominant religion.

### 4.2. Level of Self-Regulation

Self-regulation (the ability to control emotions and behavior according to the situation) within Guatemalan families is a daily practice in the urban and rural cities around the country since racism and discrimination have been a constant feature since the Spanish invasion in the 15th Century [58]. However, it is much higher in rural areas because of the values of each person are fundamental for their development from the daily life standpoint. The multiple ancestral values in each person are developed from their constant and intrinsic evolution, not leaving aside those officially offered by the state. There are asymmetrical relationships in the transmission of cultural traits between what people generate within themselves and what the government establishes; these values are developed in a traditional way (e.g., values such as dialogue and respect, which are essential for the Mayan peoples, allow the elaboration of judgments that self-regulate learning from the sociocultural reality of these people). This means that youths in Guatemala learn to control their emotions and behaviors based on the experience of their own culture (e.g., family) and those required by their community (e.g., the government).

### 4.3. Participants’ Health Literacy

Regarding emergency services, the at-risk youths in Guatemala did not report a significant attendance at health and emergency medical centers. This is a result of the fact that, in urban areas, private health costs are high; the practice of self-medication and public services can be considered precarious. In rural areas, the cultural traditions of the communities are transmitted to the youths in which local healers are consulted before governmental health services. Additionally, in the rural areas, the scarce emergency services cannot meet the demands of the population. The high rates of health literacy in rural areas is due the youths benefitting from the double impact of two cultural systems: (i) based on the information promoted by the national public health system and (ii) the information promoted by the traditional medicine of their community. Health and sports are human needs that are associated with and self-regulated by this historical knowledge within each person. In the case of health, the traditional medicine of Mayas (e.g., midwives, healers) self-regulates their values by the autochthonous knowledge despite the policies of prevention, promotion, healing, and rehabilitation of the official Western medicine. In the urban areas, these results decrease because the youths have access to other sources of health promotion, and for this reason, state policy does not focus their major efforts in this area. Participants in the sports-based intervention have not had an impact on their health awareness as a result of the programming of the organization, which is based on teaching values and not on increasing the health literacy of the participants [43,59]. The age of the youths correlated positively with health care and attention in both rural and urban areas, so it seems that the older the child, the higher the literacy in this dimension is.

### 4.4. Participants’ Physical Activity

Youths participating in the educational organization mostly practice sports when they have access to safe spaces (e.g., sports facilities, gymnasiums) provided by institutions, so they depend on them for practicing physical activity [21,60,61]. This is also a consequence of the violence and crime rates in the urban areas where they live in the city, which results in their parents not allowing them to go outside without supervision, and this limitation results in low rates of physical and health literacy [57]. While young people in rural areas perform organized physical activity (e.g., physical education) in their educational centers due to the lack of adequate sports facilities in their localities, it is important to note that most young people in rural areas do not participate in organized physical activity (e.g., sports) outside their educational centers as a result of the limited existence of youth programs in their communities. However, it is important to note that the concept of physical activity varies in the rural area, since young people who engage in activities such as walking, working agriculture, and others do so as a part of their daily lives. Self-esteem, as in other studies [62], shows that youths participating in the educational organization in urban areas have higher self-esteem, and in rural areas it is decreased. This happens because of culture-related conditions, as the youths participating in the educational organization obtain recognition for their achievements on a constant basis (e.g., academic, sports), while in the rural area, obtaining recognition is much more complicated as result of their cultural settings. Physical activity produced a higher empathy and socioemotional competence in men participating in the educational organization as a result of using and standardized educational curriculum through sports.

## 5. Conclusions

The results of the comparative study of the characteristics of youths participating in the educational organization or not showed that the level of physical activity, socioemotional competencies, and self-esteem of at-risk youths in Guatemala was especially low. However, the at-risk youths participating in the sports for education organization developed more self-esteem, and their levels of physical activity were higher than their peers not participating in the sports for education organization; the socioemotional competencies of self-regulation and motivation were higher in urban areas; empathy was higher in men than in women; the level of self-perception of health was lower and health literacy higher. In some variables of the study, rural Guatemalan youths scored better than those in urban areas. The youths not participating in the educational organization developed a set of attitudes, knowledge, skills, and abilities as result of their historical development and sociocultural strategies for health and physical activity.

The transmission of strategies takes place through human development which comprises education and cultural traits transmitted from generation to generation in rural areas from three elements: the form, function, and meaning of these traits: (i) what is visible about the trait, (ii) the need that it solves, and (iii) the ideas that are around that trait. The development of the culture of physical activity and health is established from interculturality based on the values of each of the four peoples that make up the Guatemalan nation. It is advisable not to use the concept of intervention, but to establish collaboration from a relationship at the same intercultural level in the case of countries of the Global South.

The use of physical activity (e.g., sports, physical education, and recreation) to foster the health of at-risk youths can become a good strategy for community development by establishing pedagogical tools according to the local needs for health literacy education (e.g., health services, the recognition of the symptoms of common diseases, and the prevention of non-communicable diseases) for improving the quality of life of the Guatemalan youths, especially those at-risk.

## 6. Limitations

The study design tried to neutralize the limitations of the study, mainly those affecting the generalizability of the data and researcher bias. Firstly, it must be considered that the study collected data from participants within an educational organization and non-participants residing in rural Guatemala. Secondly, although most of the participants were from rural areas, the institution under study is in an urban environment. Thirdly, we must consider the intervention of extraneous variables that could have mediated the results; therefore, the differences between the youths that participate and those that do not are not due to the effectiveness of the program, but could be a result of the influence of these variables.

## 7. Future Research

This study was exploratory in nature; it could be interesting to extend the study with qualitative data to explain the findings and help to interpret them more rigorously. Therefore, in future studies, it is intended to consider the voices of other stakeholders such as coaches, parents, or volunteers to triangulate the results and control possible variables that could influence the results. Furthermore, research could be oriented towards the analysis of similar experiences in different geographical regions that use sports as a tool to promote wider social benefits to consolidate the scientific evidence and incorporate other populations as participants in the research, which may allow the saturation of information on the subject. On the other hand, new studies could examine a broader range of issues related to conflict and violence faced by at-risk youths. The results of this study can serve as a diagnostic tool for the design of programs with groups of at-risk adolescents in Guatemala and countries with similar cultural and social characteristics, as well as the evaluation of their possible impact.

## Figures and Tables

**Table 1:** Source of family income as a function of participation in the educational organization.

Chi-Square = 0.0001	Participating: N = 90		Not Participating: N = 91	
Income	Frequency	Percentage	Frequency	Percentage
EUR 500	6	6.7	56	61.5
EUR 501–1000	72	80.0	33	36.3
EUR 1001	12	13.3	2	2.2

**Table 2:** Physical activity level, health literacy, socioemotional competencies, and self-esteem in at-risk youths in Guatemala.

	N	Min.	Max.	Median	DS	Sim.	Kurtosis
Self-esteem	181	10.00	37.00	21.508	4.33	0.057	1.288
PAQ (PAQ: Physical Activity Questionnaire for Adolescents)	181	2.00	4.78	2.878	0.530	0.823	0.425
Attention	181	−16.67	50.00	27.371	12.37	−0.695	0.997
Prevention	181	−16.67	50.00	26.697	13.24	−0.602	0.894
Promotion	181	−16.67	50.00	26.738	13.27	−0.778	1.009
HL (HL: Health Literacy)	181	−16.67	50.00	26.935	11.82	−0.778	1.489
Interpersonal regulation	181	1.67	5.00	3.352	0.639	0.235	−0.099
Motivation	181	1.00	5.00	3.677	0.717	−0.368	0.158
Self-awareness	181	1.00	5.00	3.413	0.711	−0.095	0.405
Conflict resolution	181	1.40	4.80	3.211	0.567	−0.355	0.647
Teamwork	181	0.00	4.00	2.625	0.885	−0.781	0.847
Empathy	181	0.00	4.00	2.556	0.912	−0.555	−0.058
Self-regulation	181	0.00	4.00	2.582	0.974	−0.755	0.124
SEC (SECs: Socioemotional Competence)	181	1.96	4.30	3.059	0.434	−0.036	−0.160

**Table 3:** Disparities in the amount of physical activity, health literacy, socioemotional competencies, and self-esteem between urban and rural areas.

	Area	N	Median	DS	T	Sig.	ES
Self-esteem	Urban	91	22.197	4.450	2.149	0.033	0.6452
	Rural	90	20.811	4.226			
PAQ	Urban	91	2.969	0.591	2.336	0.021	0.7788
	Rural	90	2.787	0.444			
Attention	Urban	91	24.233	14.793	−3.548	0.001	1.778
	Rural	90	30.544	8.268			
Prevention	Urban	91	24.847	16.185	−1.909	0.058	1.949
	Rural	90	28.567	9.099			
Promotion	Urban	91	24.381	15.918	−2.440	0.016	1.941
	Rural	90	29.120	9.419			
HL	Urban	91	24.487	14.077	−2.864	0.005	1.718
	Rural	90	29.410	8.362			
Interpersonal regulation	Urban	91	3.518	0.727	3.627	0.000	0.9184
	Rural	90	3.185	0.484			
Motivation	Urban	91	4.033	0.621	7.713	0.000	0.923
	Rural	90	3.318	0.624			
Self-awareness	Urban	91	3.494	0.806	1.552	0.123	0.1056
	Rural	90	3.331	0.595			
Conflict resolution	Urban	91	3.265	0.681	1.315	0.190	0.839
	Rural	90	3.155	0.418			
Teamwork	Urban	91	2.520	1.075	−1.608	0.110	0.1307
	Rural	90	2.731	0.628			
Empathy	Urban	91	2.463	1.103	−1.383	0.169	0.1349
	Rural	90	2.650	0.660			
Self-regulation	Urban	91	2.364	1.163	−3.099	0.002	0.1411
	Rural	90	2.802	0.673			
SEC	Urban	91	3.094	0.494	1.079	0.282	0.644
	Rural	90	3.024	0.363			

**Table 4:** Calculation of frequencies and association between health variables as a function of the participation in the educational organization or not of at-risk youths.

Area	No. of Hospital Admissions			Total	Chi-Squared
	1	2	3+		
Urban	47	33	11	91	0.0001
Rural	90	0	0	90	
Total	137	33	11	181	
	Emergency services				
Urban	64	24	3	91	0.214
Rural	67	23	0	90	
Total	131	47	3	181	
	Practice of physical exercise				
Urban	5	17	69	91	0.001
Rural	2	39	49	90	
Total	7	56	118	181	

**Table 5:** Pearson correlations between the different dependent variables with age.

		Age	1	2	3	4	5	6	7	8	9	10	11	12	13
Self-esteem (1)	R	0.023	1												
	Sig.	0.762													
PAQ (PAQ: Physical Activity Questionnaire for Adolescents) (2)	R	−0.050	−0.190 *	1											
	Sig.	0.504	0.011												
Attention (3)	R	0.159 *	0.047	0.012	1										
	Sig.	0.033	0.529	0.878											
Prevention (4)	R	0.084	0.092	−0.029	0.773 **	1									
	Sig.	0.263	0.220	0.694	0.000										
Promotion (5)	R	0.067	−0.023	0.098	0.707 **	0.760 **	1								
	Sig.	0.370	0.759	0.188	0.000	0.000									
HL (HL: Health Literacy) (6)	R	0.112	0.042	0.030	0.902 **	0.928 **	0.905 **	1							
	Sig.	0.134	0.574	0.691	0.000	0.000	0.000								
Interpersonal regulation (7)	R	−0.105	−0.058	0.123	−0.096	−0.025	−0.009	−0.046	1						
	Sig.	0.160	0.435	0.100	0.199	0.739	0.900	0.536							
Motivation (8)	R	−0.142	−0.068	0.084	−0.102	−0.005	0.018	−0.031	0.589 **	1					
	Sig.	0.056	0.363	0.262	0.170	0.942	0.814	0.677	0.000						
Self- awareness (9)	R	−0.004	−0.127	0.083	−0.074	−0.039	−0.010	−0.044	0.526 **	0.623 **	1				
	Sig.	0.957	0.088	0.265	0.320	0.602	0.891	0.553	0.000	0.000					
Conflict resolution (10)	R	0.050	−0.096	0.052	−0.115	−0.162 *	−0.093	−0.136	0.321 **	0.391 **	0.346 **	1			
	Sig.	0.500	0.200	0.486	0.122	0.029	0.213	0.069	0.000	0.000	0.000				
Teamwork (11)	R	0.103	0.026	0.038	0.695 **	0.735 **	0.790 **	0.813 **	−0.048	0.019	−0.007	−0.089	1		
	Sig.	0.167	0.730	0.615	0.000	0.000	0.000	0.000	0.524	0.805	0.927	0.233			
Empathy (12)	R	0.074	0.135	−0.015	0.751 **	0.874 **	0.625 **	0.822 **	−0.018	−0.033	−0.073	−0.141	0.593 **	1	
	Sig.	0.325	0.069	0.838	0.000	0.000	0.000	0.000	0.813	0.659	0.327	0.059	0.000		
Self- regulation (13)	R	0.114	0.068	−0.058	0.829 **	0.761 **	0.638 **	0.812 **	−0.086	−0.065	−0.084	−0.098	0.598 **	0.741 **	1
	Sig.	0.126	0.365	0.436	0.000	0.000	0.000	0.000	0.252	0.383	0.261	0.190	0.000	0.000	
SEC (Socioemotional Competence) (14)	R	0.042	−0.006	0.062	0.610 **	0.674 **	0.604 **	0.691 **	0.485 **	0.553 **	0.505 **	0.328 **	0.637 **	0.655 **	0.645 **
	Sig.	0.579	0.935	0.403	0.000	0.000	0.000	0.000	0.000	0.000	0.000	0.000	0.000	0.000	0.000

*, ** refers to *p* < 0.05, *p* < 0.01.

## Data Availability

The data in this study are available upon reasonable request from the corresponding author.
